# Fabrication of Magnetic Molecularly Imprinted Polymers for Selective Extraction of Dibutyl Phthalates in Food Matrices

**DOI:** 10.3390/foods13091397

**Published:** 2024-05-01

**Authors:** Lina Li, Yunzhu Lu, Chengtao Wang, Lei Cheng

**Affiliations:** Beijing Advanced Innovation Center for Food Nutrition and Human Health, Beijing Engineering and Technology Research Center of Food Additives, Beijing Technology & Business University (BTBU), Beijing 100048, China; lln18731306300@163.com (L.L.); lyz754784549@163.com (Y.L.); wct5566@163.com (C.W.)

**Keywords:** Fe_3_O_4_@MOF@MIP160, phthalate esters, molecularly imprinted polymer, magnetic solid-phase extraction

## Abstract

In this study, a novel magnetic molecularly imprinted polymeric material (Fe_3_O_4_@MOF@MIP-160) with a metal-organic backbone (Fe_3_O_4_@MOF) carrier was prepared using dibutyl phthalate (DBP) as a template. The material can be used for the efficient, rapid, and selective extraction of trace amounts of phthalic acid esters (PAEs) in food and can detect them via gas chromatography-mass spectrometry (GC-MS). The synthesis conditions of the materials were optimized to prepare the Fe_3_O_4_@MOF@MIP160 with the highest adsorption performance. Transmission electron microscopy (TEM), Fourier Transform Infrared Spectra (FT-IR), Vibration Sample Magnetic (VSM), and the Brunauer–Emmett–Teller (BET) method were used to characterize the materials. Compared with Fe_3_O_4_@MOF and the magnetic non-imprinted polymeric material (Fe_3_O_4_@MOF@NIP), Fe_3_O_4_@MOF@MIP-160 possesses the advantages of easy and rapid manipulation of magnetic materials, the advantages of high specific surface area and the stability of metal–organic frameworks, and the advantages of high selectivity of molecularly imprinted polymers. Fe_3_O_4_@MOF@MIP-160 has good recognition and adsorption capacity for di-butyl phthalate (DBP) and diethylhexyl phthalate (DEHP): the adsorption capacity for DBP and DEHP is 260 mg·g^−1^ and 240.2 mg·g^−1^, and the adsorption rate is fast (reaching equilibrium in about 20 min). Additionally, Fe_3_O_4_@MOF@MIP160 could be recycled six times, making it cost-effective, easy to operate, and time-saving as compared to traditional solid-phase extraction materials. The phthalate ester content in drinking water, fruit juice, and white wine was analyzed, with recoveries ranging from 70.3% to 100.7%. This proved that Fe_3_O_4_@MOF@MIP160 was suitable for detecting and removing PAEs from food matrices.

## 1. Introduction

Phthalate esters (PAEs) are endocrine disruptors commonly found in the environment [1]. Humans are exposed to plasticizers through diet, air inhalation, and dermal contact penetration. Thus, food products with high levels of plasticizers are hazardous to human health [2]. When added to food, plasticizers such as diethylhexyl phthalate (DEHP), di-butyl phthalate (DBP), and diisononyl phthalate (DINP) can damage human health [3]. Phthalates are added to food as they can increase flexibility, extensibility, and swelling. Consequently, exposure to these chemicals during food processing and in food packaging materials might cause plasticizer leakage and contamination [4,5]. The molecular structure of PAEs is similar to that of hormones, and they are classified as a suspected environmental hormone. Once PAEs enter the human body, due to their solubility in fats and organic solvents and insolubility in water, they will accumulate in the human body and not be easily discharged; prolonged intake will lead to high residual concentrations of PAEs in the human body. If ingested over a long period, PAEs can interfere with the secretion of human hormones, leading to abnormalities in the sex of children born to pregnant women, jeopardizing the reproductive ability of males, promoting precocious puberty in females, menstrual disorders, and infertility, and leading to other damage to the reproductive system. Moreover, some phthalates or their metabolites are suspected to be human carcinogens and endocrine disruptors [6,7,8]. Some phthalates have been included in the list of pollutants in different countries because they pose significant risks to human health and the environment [9]. PAEs are fat-soluble substances. After entering the human body, they accumulate in fatty tissues and are not easily excreted. If ingested over a long time, high levels of toxic phthalates can accumulate in the human body [10,11]. With the ever-increasing attention to food safety, the concentration of phthalates has been decreasing in food. However, food testing and analysis have serious matrix interference [12]. Thus, it is the need of the hour to develop simple, highly selective, and sensitive sample pretreatment techniques to detect PAEs in complex food matrices. The Notice on the Notification of Maximum Residue Levels of Phthalates in Foods and Food Additives issued by the General Office of the Ministry of Health of China in 2011 (Weiwei Office Supervision Letter (2011) No. 551) has already listed the permissible maximum residue in foods: the maximum residue for DBP is set at 0.3 mg·kg^−1^ and for DEHP at 1.5 mg·kg^−1^. In white spirits, the maximum acceptable concentrations for DEHP and DBP are 5 mg·kg^−1^ and 1 mg·kg^−1^, respectively.

Due to the advantages of simple operation, low cost, and ease of use, solid-phase extraction (SPE) is an important separation and enrichment technique [13]. The core of the solid-phase extraction (SPE) technique is the extraction material; this is the key factor in determining the extraction and separation efficiency. Therefore, it is essential to develop efficient and selective adsorbents. In recent times, the preparation and application of novel high-efficiency extraction materials have generated significant attention. Commonly used solid-phase extraction materials include C_8_, C_18_, cotton fiber, silica, graphitized carbon black composite, and neutral alumina. These materials adsorb targeted small molecules via molecular forces such as hydrophobicity, hydrogen bonding, π-πstacking, and ion exchange [14]. Once adsorbed, the impurities can be easily removed by washing and elution. This leads to the selective purification and enrichment of the target components [15,16,17,18,19]. Magnetic solid-phase extraction (MSPE) is convenient, adjustable, and widely used in biotechnology, medicine, and analytical chemistry [20,21,22]. In this method, magnetic nanomaterials are used as extraction materials. Typically, the magnetic cores of these materials are iron oxides. The outermost layer of the material is often modified chemically to increase its adsorption and selectivity [23].

Metal–organic frameworks (MOFs) have attracted much attention due to their large specific surface area, ultra-high porosity, and tunable pore size. However, the selectivity of the ferrous MOFs is poor, and they are unable to remove low concentrations of pollutants with specificity [24,25,26,27,28,29,30,31]. Molecularly imprinted polymers (MIPs) have been widely used to separate and enrich PAEs, as they possess specific recognition sites that are complementary to the size and shape of the template molecules [32,33,34,35,36,37]. These studies demonstrate that MOF-MIP materials have a high potential for separation and enrichment of molecules, as they combine the high selectivity of MIPs with the high adsorption capacity of MOFs.

There have been many studies on nanocomposites, such as iron oxide nanostructures (IONs) combined with graphene or its derivatives (e.g., graphene oxide and reduced graphene oxide), which hold great promise for the engineering of efficient nanocomposites [38]. It is conceivable that novel magnetic molecularly imprinted polymers with multifunctional properties can be prepared if the three concepts of metal–organic backbone materials, molecularly imprinted polymers, and magnetic separation are combined in a single system. Modification of magnetic nanoparticles (Fe_3_O_4_) with the superparamagnetic effect and modification with MOFs can increase the specific surface area of the carrier and at the same time increase the adsorption of the material to the target compounds; further modification with MIPs can improve the selectivity of the material to the targets. The resultant magnetic materials will not only exhibit high selectivity and high adsorption capacity for the target molecules but also have high magnetic responsiveness [39,40,41,42,43,44,45,46,47,48,49].

Using dibutyl phthalate (DBP) as a template, this study prepared a novel magnetic molecularly imprinted polymeric material (Fe_3_O_4_@MOF@MIP160) to detect phthalate esters. The conditions for the synthesis of Fe_3_O_4_@MOF@MIP160 were optimized. The prepared Fe_3_O_4_@MOF@MIP160 was characterized by FTIR, SEM, and nitrogen adsorption–desorption. The reusability of Fe_3_O_4_@MOF@MIP160 and the PAEs’ adsorption capacity were also investigated. Finally, the prepared Fe_3_O_4_@MOF@MIP160 was coupled with GCMS to detect PAEs in real samples, such as drinking water, fruit juice, and liquor.

This study presents the development of a novel magnetic solid-phase extraction agent through the ingenious integration of magnetic solid-phase extraction technology with advanced functional new materials. It is anticipated that this innovation will demonstrate greater potential in the composite construction and surface modification of nanomaterials, potentially revolutionizing current extraction techniques. Addressing the drawbacks of the existing national standard methods, which are often cumbersome and lack universality, this research aims to establish a more efficient, straightforward, universally applicable, and environmentally friendly detection method. The main focus is on the most critical safety concerns facing the food industry, providing a robust complement and new perspective to the current food testing systems.

## 2. Materials and Methods

### 2.1. Materials

Analytical-grade chemicals were used in this study. Di-butyl phthalate (DBP; C_16_H_22_O_4_, 99.5%), Iron (III) chloride hexahydrate, iron (II) chloride tetrahydrate, N, N-Dimethylformamide (DMF, 99.5%), and benzoic acid (99.5%) were obtained from Aladdin Reagent (Shanghai, China). PVP (Mw = 40,000) and tetrakis(4-carboxyphenyl) porphyrin (TCPP, 97%) were procured from TCI (Tokyo, Japan). Zirconyl chloride octahydrate (99.9%) was obtained from InnoChem Technology (Beijing, China). Methacrylic acid (MAA, C_4_H_6_O_2_, 99.0%), azobisisobutyronitrile (AIBN, C_8_H_12_N_4_,99.0%), and ethylene glycol dimethacrylate (EGDMA, C_10_H_14_O_4_, 99.0%) were supplied by Aladdin Chemistry (Shanghai, China). Ammonia (NH_3_·H_2_O), trisodium citrate dihydrate, methyl alcohol (MeOH, CH_4_O, 99.5%), ethanol (99.5%), and acetonitrile were obtained from Macklin (Shanghai, China). Ultrapure water (Millipore Mill-Q system, Merck Millipore, Darmstadt, Germany) was used for the experiments.

### 2.2. Synthesis

#### 2.2.1. Preparation of Fe_3_O_4_@MOF

The chemical coprecipitation method was used to synthesize the magnetic nanoFe_3_O_4_. Briefly, FeCl_3_·6H_2_O (1.329 g) and FeCl_2_·4H_2_O (0.489 g) were dissolved in deionized water (30 mL) and ethanol (30 mL) under N_2_ atmosphere and stirred mechanically at 800 rpm, followed by the addition of ammonia. After stirring for 10 min, external permanent magnets were used to collect black nanoFe_3_O_4_. Next, the Fe_3_O_4_ product was washed thrice using deionized water. The PVP powder (5×) was added to the prepared Fe_3_O_4_ and shaken at 1200 rpm in a vortex shaker for 15 h at room temperature. Then, it was washed twice with water and transferred to the DMF solution to obtain Fe_3_O_4_@PVP. Finally, Fe_3_O_4_@PVP, TCPP (30 mg·mL^−1^), ZrOCl_2_·2H_2_O (15 mg·mL^−1^), and benzoic acid (280 mg·mL^−1^) were slowly stirred at 90 °C for 6 h. Finally, the Fe_3_O_4_@PVP@MOF product was washed thrice using DMF. Finally, the Fe_3_O_4_@PVP@MOF product was stored in chromatography-grade methanol solvent [50].

#### 2.2.2. Preparation of Fe_3_O_4_@MOF@MIP

The following procedure was used to synthesize Fe_3_O_4_@PVP@MOF-based DBP-imprinted polymer. First, MAA (0.2 mmol) and DBP (0.05 mmol) were dissolved in acetonitrile (20 mL), followed by ultrasonication for 30 min. After adding Fe_3_O_4_@PVP@MOF (30 mg), EGDMA (3 mmol), and AIBN (10 mg), the obtained solution was inflated with N_2_ gas for 10 min to remove O_2_, followed by stirring at 60 °C for the next 24 h. The product was washed with a mixture of methanol and acetic acid (6:1, *v*/*v*) and redistilled water to sufficiently denature the template DBP until no template molecules were detectable, resulting in Fe_3_O_4_@MOF@MIP160. Finally, these polymers were dried at 60 °C for 12 h in an oven. NIP was prepared following the same procedures without the use of DBP.

### 2.3. Characterization of Fe_3_O_4_@MOF@MIP160

The morphology of Fe_3_O_4_@MOF@MIP160 was observed using transmission electron microscopy (TEM) using a Nippon Electron-JEOL JEM-f200 (JEOL Ltd., Tokyo, Japan) instrument. Fourier transform infrared (FTIR) spectra were recorded between 400 and 2500 cm^−1^ at 25 °C using a Thermo Fisher-Nicolet IS5 (Thermo Fisher Scientific Inc., Waltham, MA, USA). A Bruker D8 Advance X-ray diffraction (XRD) spectrometer (Scientific Technology Co. Ltd., Billerica, MA, USA), was used to obtain the XRD maps. Surface charge and particle size were determined using zeta potential determination and dynamic light scattering (DLS). The Autosorb-IQ-MP (Quantachrome Instruments, Boynton Beach, FL, USA) was used to measure the N_2_ adsorption–desorption isotherms at 77 K to assess the pore structure of the samples. The specific surface area was calculated using the Brunauer-Emmett-Teller (BET) method. A Lake Shore 8600 VSM (Lake Shore Cryotronics, Ltd., Westerville, OH, USA) vibrating sample magnetometer was used to characterize the hysteresis loops with a maximum field of 1.5 T at 25 °C [51,52].

### 2.4. Sample Preparation

Drinking water, fruit juices, and white spirits were procured from local supermarkets. Ultrasonic degassing was used for degassing all samples for 30 min, followed by filtration of the insoluble impurities using a 0.22 μm filter membrane and storage in glassware at 4 °C for further use.

The separation procedure for the analysis of food samples was as follows: 5 mL of the pre-treated sample solution was measured and mixed with 10 mg of Fe_3_O_4_@MOF@MIP160 for 20 min. It was left to stand for one minute, and then Fe_3_O_4_@MOF@MIP160 was separated from the solution by an external magnet and added to 2 mL of methanol to desorb DBP and DEHP; the supernatant enriched with the target phthalate esters was successfully separated. Finally, it was analyzed by GCMS.

### 2.5. GC-MS Analysis Parameters for the Samples

Chromatographic separation was done using a Thermo Fisher TSQ 8000 EVO (Thermo Fisher Scientific Inc., Waltham, MA, USA) gas chromatography-mass spectrometer (GC-MS) with an HP-5 ms capillary column (30 m × 250 mm × 0.25 mm) and a triple quadrupole mass spectrometer. After setting the split/unsplit injector to 260 °C, 1 μL of the sample was injected without split. The carrier gas was high-purity helium (99.999%) at a flow rate of 1.0 mL·min^−1^. The column temperature procedure was as follows: the initial column temperature was set at 60 °C and held for 1 min, followed by increasing to 220 °C at the rate of 20 °C·min^−1^ and held for 1 min. Then, the temperature was increased to 250 °C at 5 °C·min^−1^ and held for 1 min and then increased to 290 °C at 20 °C·min^−1^ and held for 7.5 min.

Using the standard isotope internal standard method, initially, a standard stock solution at a concentration of 10 μg·mL^−1^ and a deuterated internal standard stock solution at the same concentration were prepared. Subsequently, a series of standard working solutions were formulated by accurately pipetting the two-phthalate ester standard stock solutions (10 μg·mL^−1^) and sequentially diluting them with chromatography-grade ethyl acetate to achieve concentrations of 10, 25, 50, 100, 250, 500, 1000, 2000, 3000, 4000, and 5000 ppb. Concurrently, a deuterated internal standard stock solution (10 μg·mL^−1^) was added to ensure that the internal standard concentration was 100 ppb for the standard series solutions ranging from 10 to 1000 ppb, and 1000 ppb for the standard series solutions ranging from 1000 to 5000 ppb. These solutions were prepared immediately before use. The proposed method was validated in terms of linearity, limit of detection (LOD), and limit of quantitation (LOQ). Every sample was tested three times by GC-MS to obtain the standard deviation. These results were analyzed by Anova software (SPSS version 22.0).

### 2.6. Dynamic Binding Experiments

By altering the synthetic conditions of the material (the ratio of template molecules to functional monomers, the ratio of template molecules to EGDMA, and the reaction time), a mixture of 4 mL PAEs (DBP and DEHP) at a concentration of 100 mg·L^−1^ was adsorbed onto 10 mg of Fe_3_O_4_@MOF@MIP160 material to ascertain the impact of these regulatory factors on the adsorption capacity. Additionally, multiple washing cycles were conducted to confirm the number of washes required to completely remove the template molecules.

The adsorption capacity for two phthalate acid esters (PAEs) was determined through adsorption kinetics experiments using the prepared Fe_3_O_4_@MOF@MIP160. Initially, 10 mg of Fe_3_O_4_@MOF@MIP160, Fe_3_O_4_@MOF@NIP, and Fe_3_O_4_@MOF adsorbents was immersed in 4 mL of a phthalate solution at a concentration of 100 mg·L^−1^. After allowing for adsorption for 2, 4, 6, 8, 10, 12, 14, 16, 18, 20, 22, 24, 26, 28, and 30 min, the supernatant was taken for subsequent analysis. The concentrations of dibutyl phthalate (DBP) and di(2-ethylhexyl) phthalate (DEHP) in the post-adsorption solution were measured using gas chromatography-mass spectrometry (GC-MS). The following equations were employed to calculate the adsorption capacity (Qe, milligrams per gram) and the recovery rate (R, %), as shown in (1) and (2):

(1) $$Q_{e}=\frac{(C_{0}-{\;C}_{t})\times V}{m}$$

(2) $$R(\%)=\frac{C_{0}-{\;C}_{t}}{C_{0}}\times 100$$

where *V* represents the volume of PAE solution (mL); *C*_0_ and *C_t_* indicate the initial and real-time concentrations of the PAE solution (mg·L^−1^), respectively; *Q_e_* (mg·g^−1^) represents the amount of PAEs adsorbed by the adsorbent; and m is the mass of Fe_3_O_4_@MOF@MIP160 and Fe_3_O_4_@MOF@NIP.

### 2.7. Stability Experiment

To examine the stability of the materials, 10 mg of Fe_3_O_4_@MOF@MIP160 was removed from the magnetic material stock solution as an extractant for the adsorption of the two phthalate esters at 15-day intervals for 45 days of continuous experiments.

### 2.8. Selective Research

Selective research was conducted by adding known levels of common interfering components in foods (vitamin C, whey protein, calcium ions) as well as benzylamine under the simulation system. Benzylamine is an aromatic amine compound that may be found in foods and has a structure similar to PAEs. The above four substances were utilized to explore the adsorption selectivity properties of the composites for the targets.

### 2.9. Regeneration of Adsorption of Fe_3_O_4_@MOF@MIP160

Fe_3_O_4_@MOF@MIP160 (10 mg) was suspended in 4 mL of a methanol solution of phthalates (50 mg·L^−1^). The adsorption process was conducted as follows: initially, 10 mg of Fe_3_O_4_@MOF@MIP160 adsorbent was immersed in 4 milliliters of a phthalate ester solution with a concentration of 100 mg·L^−1^. After 20 min of adsorption, which allowed the system to reach adsorption equilibrium, the supernatant was collected for further analysis. After adsorption, the material was washed thrice with methanol and dried at 60 °C until constant weight was obtained. This adsorption–desorption cycle was repeated six times.

## 3. Results

### 3.1. Optimization of the Synthesis Conditions

For magnetic composites, the core material must maintain its superparamagnetism. Thus, the conditions for the synthesis of Fe_3_O_4_@MOF@MIP160 were optimized to achieve the best adsorption performance of the composites.

#### 3.1.1. Effect of the Ratio of Template Molecules to Functional Monomer

**Figure 1:**
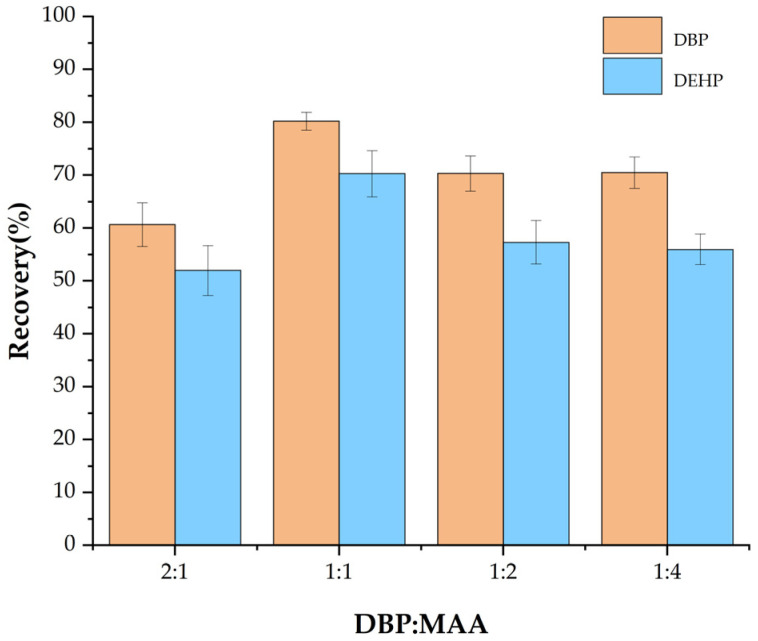
Effect of the ratio of template molecule to functional monomer on the adsorption performance of Fe_3_O_4_@MOF@MIP160.

The imprinting efficiency and affinity of MIP depend on the molar ratio between the template (DBP) and the functional monomer (MAA). This study examined the effect of three different ratios of DBP to MAA—2:1, 1:1, 1:2, and 1:4—on the PAE adsorption performance of Fe_3_O_4_@MOF@MIP160 (Figure 1). When the DBP to MAA ratio was 2:1, there was not enough monomer to form a complete recognized cavity, resulting in low recovery. With the increase of the monomer ratio, the adsorption capacity of Fe_3_O_4_@MOF@MIP160 gradually enhanced. The highest recoveries (DBP: 80.2%, DEHP: 70.3%) were obtained for Fe_3_O_4_@MOF@MIP160 when the ratio reached 1:1. This might be because the ester group on DBP formed a complete complex with the hydrogen on the amino group in MAA, resulting in a complete recognition cavity. When the DBP-to-MAA ratios were 1:2 and 1:4, the recovery was lower due to the decrease in the number of templates, which, in turn, decreased the amount of effective MAA–DBP complexes. Since there were not enough MAA–DBP complexes, the overall selectivity of the MIP was reduced. Thus, 1:1 was chosen as the optimal ratio of template molecule to functional monomer.

#### 3.1.2. Effect of the Ratio of Template Molecules to EGDMA

**Figure 2:**
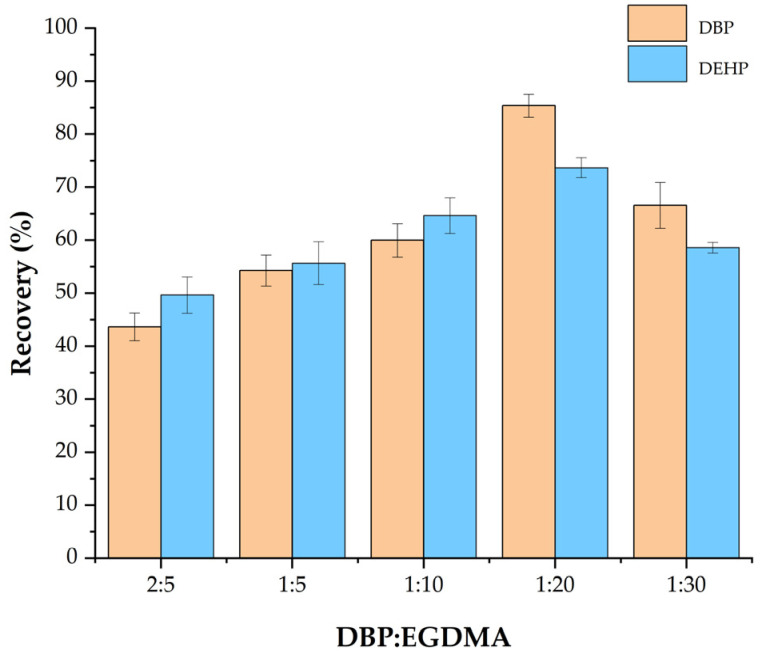
Effect of the ratio of template molecules to EGDMA on the adsorption performance of Fe_3_O_4_@MOF@MIP160.

The content of EGDMA, a cross-linking agent, impacts the polymer network structure as well as the polarity of the adsorbent. This affects the recognition and adsorption of the test substance on Fe_3_O_4_@MOF@MIP160. Therefore, the adsorption properties of Fe_3_O_4_@MOF@MIP160 were examined at the following DBP-to-EGDMA ratios: 2:5, 1:5, 1:10, 1:20, and 1:30 (Figure 2). The highest recovery rate (DBP: 73.7%, DEHP: 85.4%) was observed at the DBP–EGDMA of 1:20. At the other ratios, i.e., 2:5, 1:5, and 1:10, the lack of EGDMA and the inability of MIP to form a stable pore structure resulted in a high rate of MIP loss. In contrast, when the ratio was 1:30, the recovery rate decreased because of the presence of excessive EGDMA. This led to a denser polymer network structure in Fe_3_O_4_@MOF@MIP160, which increased the spatial site resistance of the molecularly imprinted sites. Therefore, 1:20 was chosen as the optimal template molecule-to-EGDMA ratio.

#### 3.1.3. Effect of Reaction Time

**Figure 3:**
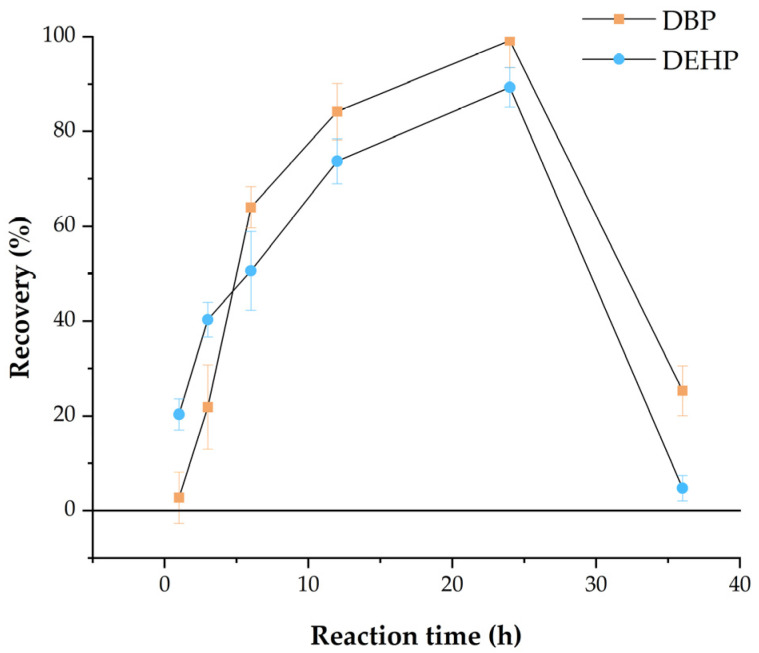
Effect of reaction time on the preparation of Fe_3_O_4_@MOF@MIP160.

The effectiveness of magnetic material preparation, as well as the adsorption properties of the composites, are affected by reaction time. The adsorption properties of Fe_3_O_4_@MOF@MIP160 were examined at the following reaction times: 1, 3, 6, 12, 24, and 36 h. As shown in Figure 3, the highest recovery (DBP: 99.2%, DEHP: 89.33%) was obtained when the reaction time was 24 h. At reaction times < 24 h, the molecular imprinting polymerization was incomplete, while at reaction times > 24 h, the product color was darker brown due to oxidation that may have occurred, leading to poor binding.

#### 3.1.4. Number of Washes Required to Completely Remove Stencil Molecules

The last step requires the removal of the stencil molecules from the product to finally obtain the molecularly imprinted polymerization with a precise 3D imprinted cavity structure, which is complementarily matched with the template molecules in terms of geometric morphology, dimensions, and layout of the functional groups to ensure a high degree of selectivity for the target molecules, as well as to expand the adsorption capacity and improve the adsorption ability of the material.

**Figure 4:**
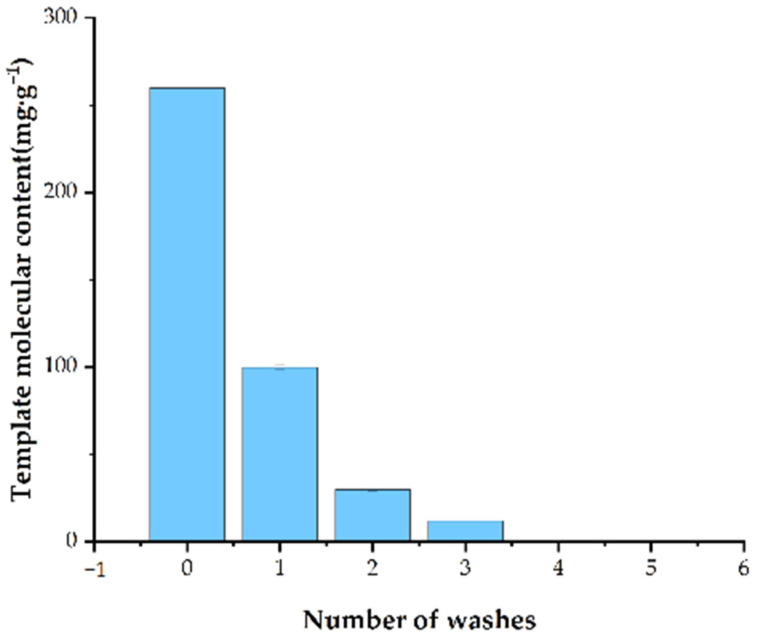
The number of washes required to completely remove stencil molecules.

To ensure that no template molecules remained in the fabricated Fe_3_O_4_@MOF@MIP160, the template molecule DBP was denatured by washing with methanol and acetic acid (6:1, *v*/*v*) and distilled water several times until no template molecules were detected. As shown in Figure 4, the results showed that most of the template molecules had been removed by the second wash, and there were no template molecules left at all by the fourth wash, so the final choice was to wash four times.

### 3.2. Characterization of Adsorbent Materials

#### 3.2.1. TEM, DLS, and Zeta Analysis

**Figure 5:**
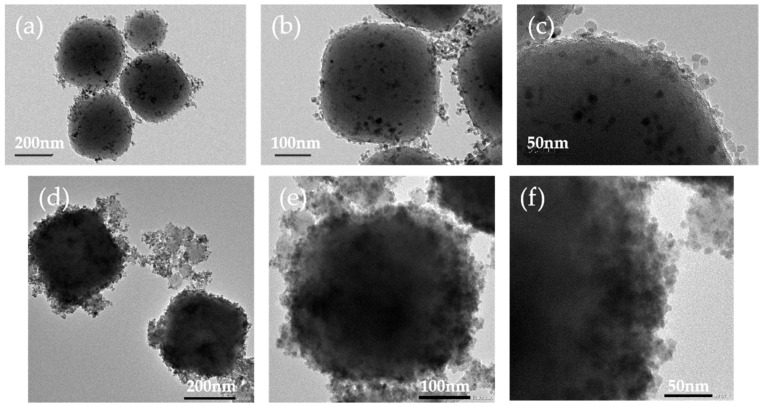
TEM images of (**a**–**c**) Fe_3_O_4_@MOF; (**d**–**f**) Fe_3_O_4_@MOF@MIP160.

TEM was used to characterize the morphology and structure of the material (Figure 5).

**Figure 6:**
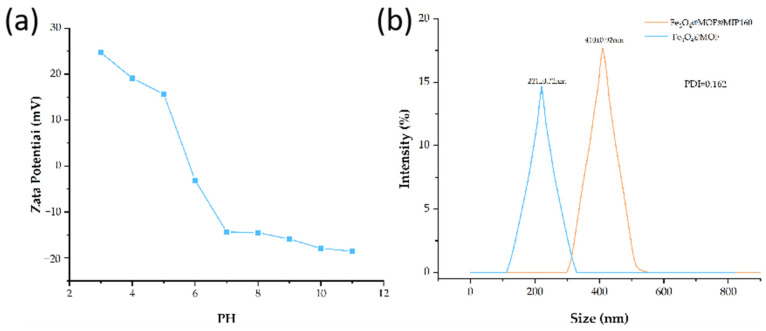
(**a**) Zeta potential; (**b**) DLS testing of Fe3O4@MOF and Fe_3_O_4_@MOF@MIP160.

The results showed that the nanospheres are monodisperse irregular spheres with uniform particle size. Figure 5e,f shows that the surface of Fe_3_O_4_@MOF@MIP160 is rough, with many irregular deposits around it, which are considered imprinted polymers, suggesting that the MIP has been covered on the surface of Fe_3_O_4_@MOF in the form of a thin layer. The TEM results showed that the particle size of Fe_3_O_4_@MOF is in the range of 200–210 nm and Fe_3_O_4_@MOF@MIP160 is in the range of 350–400 nm. As shown in Figure 6b, the particle size of the nanoparticles was then characterized by DLS. The uncoated nano-Fe_3_O_4_@MOF has a uniform size distribution with an average diameter of (221 ± 0.32) nm, and the coated nano-Fe_3_O_4_@MOF@MIP160 shows a uniform size distribution with an average diameter of (410 ± 0.92) nm. The MIP layer was uniformly dispersed on the surface of Fe_3_O_4_@MOF, and the thickness of the MIP layer deposited on the surface of Fe_3_O_4_@MOF was about 95 nm. This structure accelerates the rate of adsorption and facilitates the adsorption of target molecules. The particle size results for TEM were slightly smaller than the DLS test; this was because the TEM test samples of nanoparticles were dried, while the DLS test was carried out in solution. Since the surfactant swells in liquids, the DLS test particle size data were generally slightly larger. The polymer dispersibility index (PDI) was 0.162, which shows good dispersibility. The results of the Zeta analysis are shown in Figure 6a, demonstrating that the prepared material has a large positive and negative potential span (43.2 mV), indicating good stability.

#### 3.2.2. XRD Analysis

**Figure 7:**
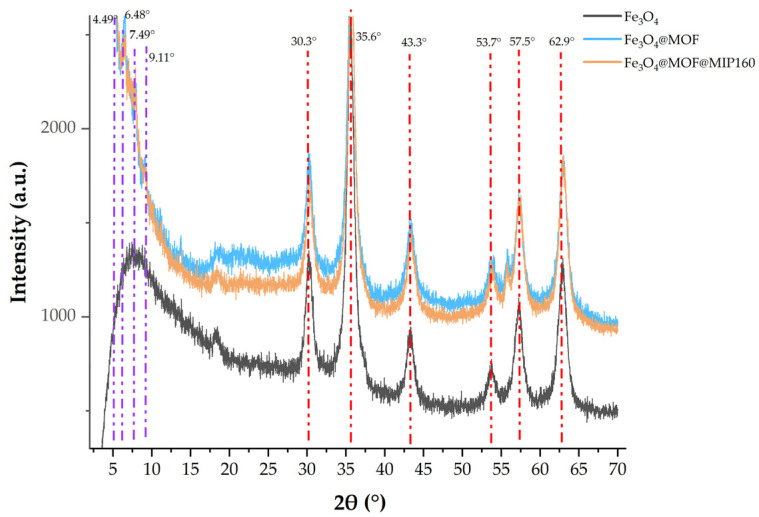
Wide-angle XRD patterns of Fe_3_O_4_, Fe_3_O_4_@MOF, and Fe_3_O_4_@MOF@MIP160.

The synthesized crystal structures and phase purities were further investigated by characterizing the X-ray single crystal diffraction (XRD) of Fe_3_O_4_, Fe_3_O_4_@MOF, and Fe_3_O_4_@MOF@MIP160 (Figure 7). The peaks at 2 θ = 30.3, 35.6, 43.3, 53.7, 57.5, and 62.9 can be assigned to the (220), (311), (400), (422), (511), and (440) characteristic diffraction peaks of the Fe_3_O_4_ nanoparticles, respectively (JCPDS no. 19-629). Comparison of XRD diffraction analysis of Fe_3_O_4_@MOF revealed that no impurity peaks were found, except for the crystalline Fe_3_O_4_ and MOF peaks (at 4.54°, 6.44°, 7.88°, and 9.14°), which indicated that the direct crystallization degree of the two was good and the incorporation of the MOF material did not destroy the original crystal structure of Fe_3_O_4_. No significant difference was found between Fe_3_O_4_@MOF@MIP160 and Fe_3_O_4_@MOF.

#### 3.2.3. VSM Analysis

**Figure 8:**
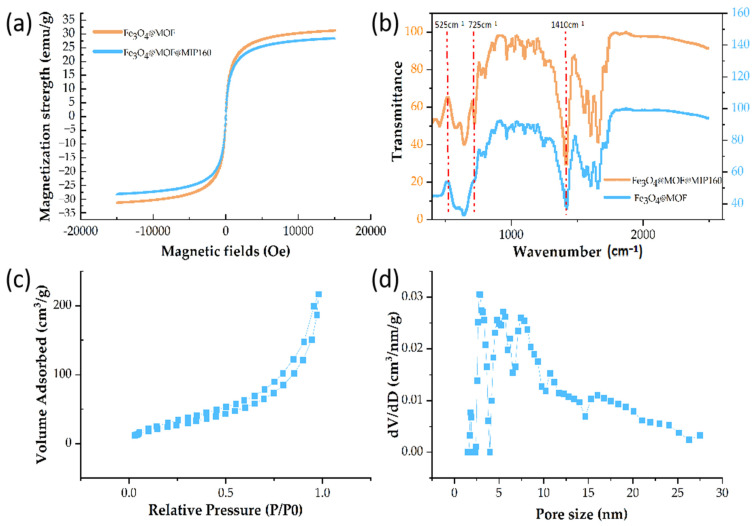
(**a**) Hysteresis curves of Fe_3_O_4_@MOF and Fe_3_O_4_@MOF@MIP160; (**b**) FTIR spectra of Fe_3_O_4_@MOF and Fe_3_O_4_@MOF@MIP160; (**c**,**d**) N_2_ adsorption isotherm and pore size distribution curve of Fe_3_O_4_@MOF@MIP160.

Magnetic field properties are crucial for separating magnetic materials. The saturation magnetization strength of Fe_3_O_4_ was around 60.7 emu·g^−1^. The VSM method was used to determine the magnetic properties of Fe_3_O_4_@MOF and Fe_3_O_4_@MOF@MIP160. The application and the magnetic field magnetization intensity of the materials were plotted. The magnetization saturation values of Fe_3_O_4_@MOF and Fe_3_O_4_@MOF@MIP160 were 30.12 emu·g^−1^ and 27.06 emu·g^−1^, respectively (see Figure 8a). Moreover, there was no observable magnetic hysteresis in the hysteresis curves, which was indicative of the excellent superparamagnetic properties of the materials. The degree of magnetization of both materials was lower than Fe_3_O_4_ because of the modification of MOF. Due to the assembly of MIPs, Fe_3_O_4_@MOF@MIP160 was found to be slightly less magnetized than Fe_3_O_4_@MOF. However, it was of sufficient size to be quickly and effectively separated by an external magnet. The results revealed that the nanocomposites have a higher separation efficiency and can be used for magnetic solid-phase extraction.

#### 3.2.4. FT-IR Analysis

The characteristic functional groups on the surface of the prepared nanocomposites were identified by FT-IR (Figure 8b). The spectrum of Fe_3_O_4_@MOF@MIP160 retained several characteristic absorption peaks of Fe_3_O_4_@MOF, such as at 1410 cm^−1^ (-C-O stretching vibration). Additionally, the absorption band at 525 cm^−1^ was attributed to the Fe-O stretching vibration, suggesting that the metal center ion was coordinated with the organic ligand. The spectrum of Fe_3_O_4_@MOF@MIP160 showed a new characteristic absorption peak at 725 cm^−1^, which could be attributed to a = C-H bending vibration arising from the polymerization between EGDMA and the benzene ring. This is because the MIP layer was grafted on the Fe_3_O_4_@MOF surface.

#### 3.2.5. Nitrogen Adsorption–Desorption

In addition to superparamagnetism, the extraction capability is also of great importance for magnetic solid-phase extractants. Notably, extraction performance is closely related to the specific surface area and pore size of the material. Both these properties can be quantitatively estimated by N_2_ adsorption, and desorption experiments can be used. Figure 8c,d shows that the Fe_3_O_4_@MOF@MIP160 adsorption isotherm belongs to the I-type isotherm. At low pressure, the composite rapidly adsorbed nitrogen to reach equilibrium, suggesting that the initial adsorption is dominated by small-sized pores. Once the system reached equilibrium, capillary condensation or multimolecular layer adsorption occurred, indicating that the material had a microporous or mesoporous structure. The specific surface area and total pore volume of Fe_3_O_4_@MOF@MIP160 were calculated to be 95.604 m^2^·g^−1^ and 0.292 cm^3^·g^−1^, respectively. Using density-functional-transfer-theoretic (DFT) calculations, the average pore size of the material was estimated to be 1.40 nm in the N_2_ isotherm. Thus, the nanocomposite’s high specific surface area and high pore size provided more active sites for adsorbing target substances. On the other hand, the small pore size structure favored the high selectivity of small molecules.

### 3.3. Analysis of Adsorption Performance of Fe_3_O_4_@MOF@MIP160

**Figure 9:**
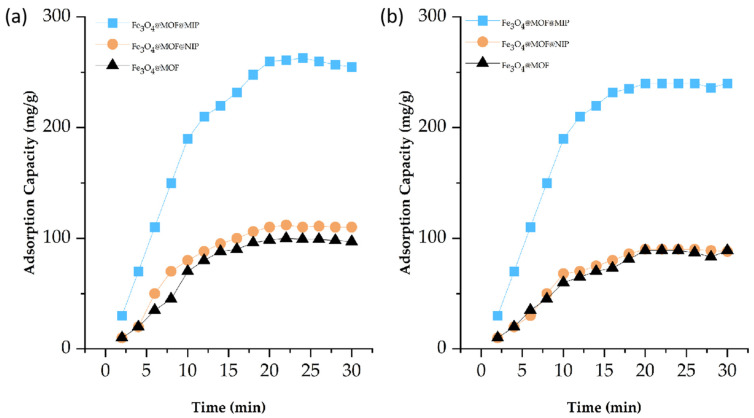
Adsorption curves of Fe_3_O_4_@MOF@MIP160, Fe_3_O_4_@MOF@NIP, and Fe_3_O_4_@MOF on DBP (**a**) and DEHP (**b**).

Figure 9 shows the dynamic adsorption curves of PAEs adsorbed on Fe_3_O_4_@MOF@MIP160, Fe_3_O_4_@MOF@NIP, and Fe_3_O_4_@MOF. The adsorption of PAEs on Fe_3_O_4_@MOF@MIP160 occurred rapidly in the first 10 min. After the first 10 min, the rate of increase slowed down, and adsorption equilibrium was reached after 20 min. The adsorption behaviors of Fe_3_O_4_@MOF@NIP and Fe_3_O_4_@MOF were similar. However, the adsorption capacity of Fe_3_O_4_@MOF@MIP160 (260.2 mg·g^−1^) for DBP was significantly higher than Fe_3_O_4_@MOF@NIP (111.4 mg·g^−1^) and Fe_3_O_4_@MOF (98.5 mg·g^−1^). Fe_3_O_4_@MOF@MIP160 (240.2 mg·g^−1^) also displayed a significantly higher adsorption capacity for DEHP than Fe_3_O_4_@MOF@NIP (90.4 mg·g^−1^) and Fe_3_O_4_@MOF (89.0 mg·g^−1^). The adsorption ability of the magnetic materials was improved by the presence of molecularly imprinted sites, especially in the selective recognition of the template molecule DBP. This is because the binding of molecularly imprinted sites best matches the size and shape of DBP. Moreover, the structure of DEHP is similar to that of DBP, resulting in better selective recognition. This suggests that specific imprinted recognition sites on the surface of Fe_3_O_4_@MOF@MIP160 match the DBP well in terms of size and spatial arrangement. The high specific surface area of the magnetic Fe_3_O_4_@MOF material also contributes to its large adsorption capacity.

### 3.4. Stability of Fe_3_O_4_@MOF@MIP160

**Figure 10:**
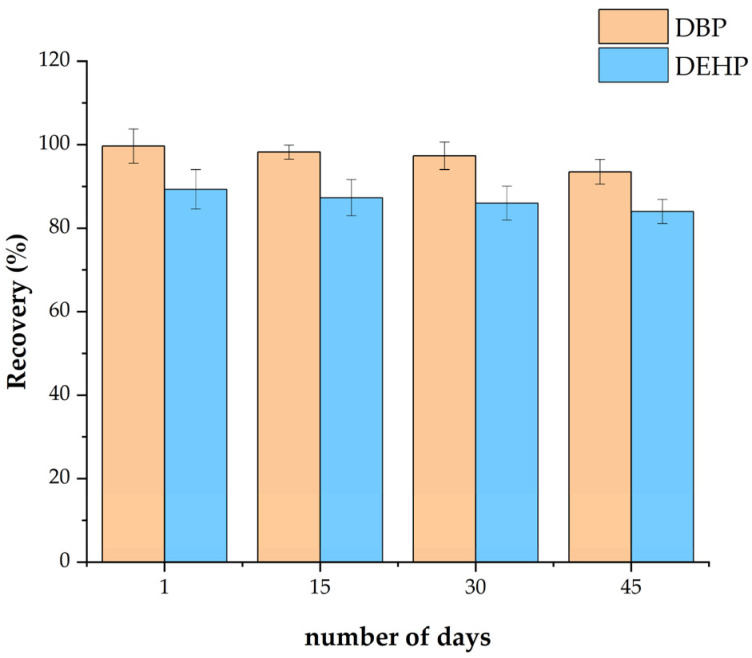
Effect of storage time on the adsorption of phthalate esters by Fe_3_O_4_@MOF@MIP160.

To verify the stability of the fabricated composite material Fe_3_O_4_@MOF@MIP160, we impregnated the synthesized samples in methanol solvent and kept them in storage for up to 45 days. An appropriate amount of adsorbent material was extracted every two weeks to examine whether its extraction efficacy for the target analytes was stable at different time intervals. As shown in Figure 10, the adsorption rate was unchanged and remained above 93% (DBP) and 84% (DEHP) after 45 days, which indicates that the material has strong stability.

### 3.5. Selectivity of Fe_3_O_4_@MOF@MIP160

**Figure 11:**
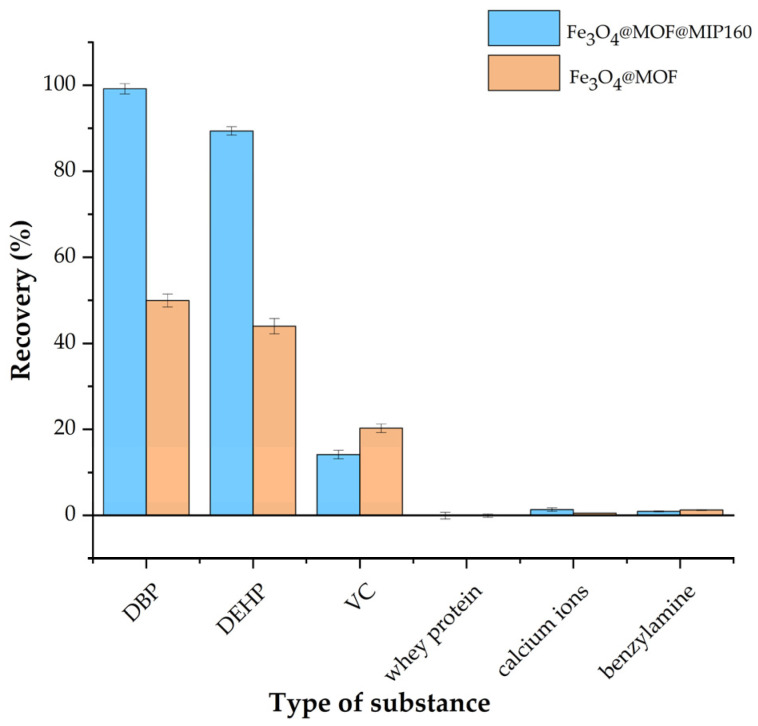
Adsorption effects of magnetic molecularly imprinted polymeric material Fe_3_O_4_@MOF@MIP160 and magnetic non-molecularly imprinted polymeric material Fe_3_O_4_@MOF@NIP on coexisting components in foods.

Because of the complexity of food matrices, it is important to consider the possible interfering effects of other co-existing substances when selecting magnetic solid-phase extraction agents and to ensure that the molecularly imprinted polymers (MIPs) used demonstrate excellent selectivity. Therefore, the adsorption properties of the resulting materials for the target compounds were systematically examined by introducing known concentrations of interfering components into the simulated system. As shown in Figure 11, the results indicate that the adsorption efficiency of the synthesized Fe_3_O_4_@MOF@MIP160 for the target compound phthalate was not significantly suppressed under the complex matrix conditions, and the highly selective adsorption of the target was still maintained. Moreover, it can be seen that the novel magnetic molecularly imprinted polymeric material (Fe_3_O_4_@MOF@MIP160) prepared with dibutyl phthalate (DBP) as a template has a higher adsorption rate for phthalate than the non-imprinted polymeric material (Fe_3_O_4_@MOF@NIP) prepared without a template and achieves a better exclusion of the common interfering components of foodstuffs, including vitamin C, whey protein, calcium ions, benzoyl methane, and benzylamine. This suggests that Fe_3_O_4_@MOF@MIP160 is selective for phthalates and can be used to extract target phthalates without interference in complex food matrices.

### 3.6. Regeneration of Fe_3_O_4_@MOF@MIP160

**Figure 12:**
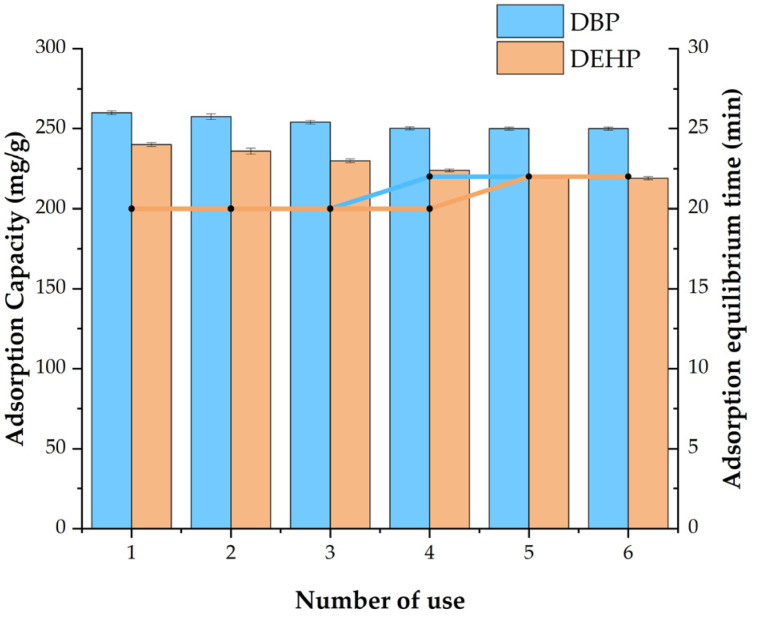
Reuse results of Fe_3_O_4_@MOF@MIP160 over six times.

The regeneration of Fe_3_O_4_@MOF@MIP160 is important to make the material cost-effective and increase its applications. As can be seen in Figure 12, the adsorption capacity of Fe_3_O_4_@MOF@MIP160 for PAEs did not change after six adsorption–desorption cycles; adsorption equilibrium time was also basically maintained at about 20 min. Thus, compared with the traditional SPE materials, Fe_3_O_4_@MOF@MIP160 can result in significant cost savings in sample pretreatment. Moreover, these materials possess a high magnetization strength and can expedite the sample pretreatment process.

### 3.7. GC-MS Methodology Evaluation

**Figure 13:**
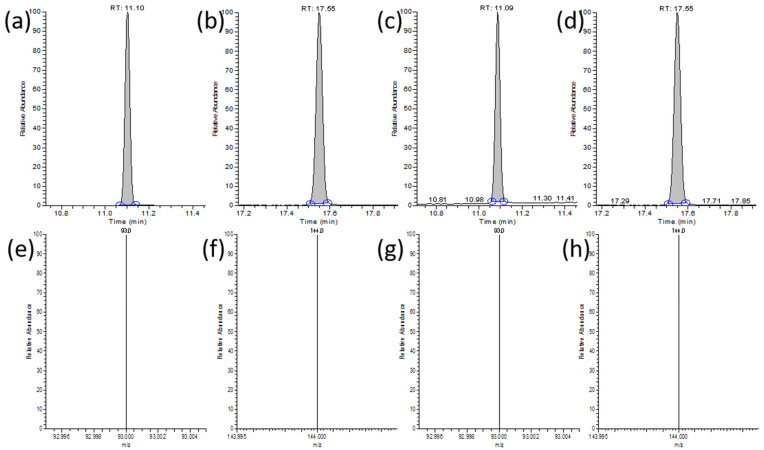
(**a**) Chromatogram of DBP in standard solution; (**b**) chromatogram of DEHP in standard solution; (**c**) chromatogram of DBP in food samples; (**d**) chromatogram of DEHP in food samples (The blue circle represents the selection range of the chromatographic peak area region); (**e**) mass spectra of DBP in standard solution; (**f**) mass spectra of DEHP in standard solution; (**g**) mass spectra of DBP in food samples; (**h**) mass spectra of DEHP in food samples.

We optimized the chromatographic and mass spectrometric parameters (Figure 13).

The respective working curves were constructed for drinking water, fruit juice, and white spirit samples. Considering the wide concentration distribution range of the target in the samples and to achieve more accurate quantitative determination, we adopted the strategy of segmented standard curves for quantitative analysis in the ranges of 10–1000 ppb and 1000–5000 ppb, respectively. The results of the standard curves as well as the correlation coefficients are shown in Table 1; the results indicate that all samples have a good linear relationship with the correlation coefficient (R^2^) ≥ 0.9991.

The limits of detection (LOD) and quantification (LOQ) of DBP in drinking water, carbonated beverages, fruit juices, and white wine samples were calculated based on the signal-to-noise ratios of analyte concentrations of 3:1 and 10:1, as well as the limits of detection (LOD) and quantification (LOQ) of DEHP. The results are shown below: The limits of detection (LOD) and quantification (LOQ) for DBP in drinking water were 0.091 µg·L^−1^ and 0.08 µg·L^−1^, respectively; for DEHP in drinking water, the LOD and LOQ were 0.13 µg·L^−1^ and 0.18 µg·L^−1^, respectively. The limits of detection (LOD) and quantification (LOQ) for DBP in fruit juice were 0.01 µg·L^−1^ and 0.06 µg·L^−1^, respectively; for DEHP in fruit juice, the LOD and LOQ were 0.04 µg·L^−1^ and 0.08 µg·L^−1^, respectively. The limits of detection (LOD) and quantification (LOQ) for DBP in white spirits were 0.06 µg·L^−1^ and 0.07 µg·L^−1^, respectively; for DEHP in white spirit, the LOD and LOQ were 0.01 µg·L^−1^ and 0.05 µg·L^−1^, respectively.

In this study, the accuracy of the proposed method was verified by performing spiked recovery experiments in three blank food sample matrices—drinking water, fruit juice, and white wine—by adding three gradients (high, medium, and low) of standard solutions to explore the recovery of target substances in each matrix. In addition, the precision was determined by investigating the relative standard deviation (RSD) of the measurements. The results are shown in Table 2. The results showed that the extraction rate was mostly in the range of 80–100% for both phthalates tested at several different spiked levels with good precision and accuracy.

In conclusion, the proposed method is suitable for the quantitative analysis and detection of phthalates in a wide range of food samples.

### 3.8. Application to Real Sample Analysis

In this study, three common commercially available beverages, i.e., drinking water, fruit juice, and white wine, were analyzed and tested. Different concentration levels of DBP and DEHP were successfully identified in the above samples, and the results are shown in Table 3. Comparison of the experimental results with the national maximum residue limits (MRLs) showed that all the detected levels of DBP and DEHP were significantly lower than the national limits, thus confirming that the levels of these two phthalate esters in the commercially available beverages were within the national safety limits.

## 4. Conclusions

By combining the high specific surface area, ultra-high porosity, and excellent stability of metal–organic frameworks (MOFs) with the specific recognition of the molecularly imprinted polymer MIP, this study prepared a novel surface molecularly imprinted polymer, Fe_3_O_4_@MOF@MIP160. First, the synthesis of Fe_3_O_4_@MOF@MIP160 was standardized by optimizing the ratio of template molecule to functional monomer, the ratio of template molecule to EGDMA, and the reaction time. The prepared Fe_3_O_4_@MOF@MIP160 displayed an excellent adsorption performance, with 99.2% recovery of DBP and 89.33% recovery of DEHP. The prepared nanocomposites were characterized by TEM, FT-IR, VSM, and BET. The results confirmed that the molecularly imprinted polymers were successfully immobilized on the surface of Fe_3_O_4_@MOF. Fe_3_O_4_@MOF@MIP160 demonstrated good specific recognition, large adsorption capacity, and sufficient magnetization strength. Further, the material showed good performance even after six cycles, suggesting that the cost of sample pretreatment during detection would be significantly lesser than traditional national standard SPE columns. The magnetic properties of Fe_3_O_4_@MOF@MIP160 would also simplify the operation steps and greatly save the pretreatment time. The nanocomposites were also used to determine phthalate esters in drinking water, fruit juices, and white wine with high accuracy and precision. The proposed method established in this study greatly simplifies the sample processing steps, saves time and costs in experiments, and is more environmentally friendly than the traditional method, while ensuring the accuracy and precision of the experiment. The findings of this study indicate that the combination of surface-imprinted polymers and magnetic composites has great potential for enriching and separating trace pollutants.

In recent years, the field of food safety testing has persistently explored methods for determining the levels of phthalate esters in the food supply, with innovative developments in novel magnetic solid-phase extraction (MSPE) techniques providing new solutions to these challenges. Within this suite of detection processes, the steps of extraction play a pivotal role. However, traditional techniques often come with significant drawbacks, including high costs, complex operational procedures, extended processing times, and environmental pollution due to the extensive use of organic solvents.

As a result, research into the composite construction and surface modification of nanomaterials has become increasingly valued. It is anticipated that these new materials will demonstrate enhanced adsorption capabilities, potentially transforming current extraction methodologies and thus improving the efficiency and environmental sustainability of food safety testing. It is foreseeable that by integrating materials with individual properties in order to modifying them, composite materials with comprehensive and superior performance can be obtained. Combining the enrichment techniques of these novel materials with sophisticated detection equipment is likely to establish a more effective detection system for trace substances in the food supply. This would render the detection methods more accurate, environmentally benign, time-saving, and cost-effective, presenting a promising prospect for the future.

## Figures and Tables

**Table 1:** Standard curve equations and correlation coefficients for different ranges in different foods.

Range		DBP		DEHP	
		10–1000 ppb	1000–5000 ppb	10–1000 ppb	1000–5000 ppb
Drinking water	standard curve	y = 0.432x + 0.562	y = 1.22x + 0.322	y = 0.426x + 0.228	y = 1.82x + 0.237
	R^2^	0.9998	0.9999	0.9991	0.9993
Fruit juice	standard curve	y = 0.335x + 0.340	y = 0.763x + 0.036	y = 0.662x + 0.716	y = 0.613x + 0.028
	R^2^	0.9998	0.9997	0.9992	0.9991
White spirits	standard curve	y = 0.55216x + 4.046	y = 0.287x + 0.887	y = 0.629x + 1.22	y = 3.27x + 0.008
	R^2^	0.9993	0.9996	0.9991	0.9991

**Table 2:** Recoveries of real samples spiked with PAEs (n = 3).

Sample	Analyte	Added (0.1 μg/L)		Added (1 μg/L)		Added (10 μg/L)	
		Extraction Rate (%)	RSD (%)	Extraction Rate (%)	RSD (%)	Extraction Rate (%)	RSD (%)
Drinking water	DBP	93.5 ± 2.6	2.4	90.9 ± 1.9	2.4	100.7 ± 3.4	3.37
	DEHP	85.3 ± 2.4	1.7	87.3 ± 1.4	2.2	90.3 ± 2.5	2.34
Fruit juice	DBP	90.8 ± 1.3	3.2	90.1 ± 1.2	1.9	97.7 ± 2.1	2.18
	DEHP	80.3 ± 2.1	1.4	81.3 ± 1.6	3.7	87.3 ± 1.5	3.16
White spirits	DBP	80.8 ± 2.3	1.5	86.1 ± 1.2	3.28	97.3 ± 3.1	1.29
	DEHP	70.3 ± 2.1	2.7	78.3 ± 1.9	2.45	87.3 ± 1.0	1.96

**Table 3:** DBP and DEHP content in actual beverage samples.

	Sample 1	Sample 2	Sample 3	Sample 4 (White Spirits)
DBP (mg/kg)	0.01	0.19	0.09	0.71
DEHP (mg/kg)	0.82	0.66	1.02	3.22

## Data Availability

The original contributions presented in the study are included in the article, further inquiries can be directed to the corresponding author.

## References

[1] Chen M., Niu Z., Zhang X., Zhang Y. (2024). Pollution Characteristics and Health Risk of Sixty-Five Organics in One Drinking Water System: PAEs Should Be Prioritized for Control. Chemosphere.

[2] Kato K., Silva M.J., Reidy J.A., Hurtz D., Malek N.A., Needham L.L., Nakazawa H., Barr D.B., Calafat A.M. (2004). Mono(2-Ethyl-5-Hydroxyhexyl) Phthalate and Mono-(2-Ethyl-5-Oxohexyl) Phthalate as Biomarkers for Human Exposure Assessment to Di-(2-Ethylhexyl) Phthalate. Environ. Health Perspect..

[3] Li X., Wang Q., Jiang N., Lv H., Liang C., Yang H., Yao X., Wang J. (2023). Occurrence, Source, Ecological Risk, and Mitigation of Phthalates (PAEs) in Agricultural Soils and the Environment: A Review. Environ. Res..

[4] Fasano E., Bono-Blay F., Cirillo T., Montuori P., Lacorte S. (2012). Migration of Phthalates, Alkylphenols, Bisphenol A and Di(2-Ethylhexyl)Adipate from Food Packaging. Food Control..

[5] Sun Q., Zhang X., Liu C., Nier A., Ying S., Zhang J., Zhao Y., Zhang Y., Wang Z., Shi M. (2023). The Content of PAEs in Field Soils Caused by the Residual Film Has a Periodical Peak. Sci. Total Environ..

[6] Gong J., Yi X., Liang J., Liu Z., Du Z. (2024). Inhibitory Effects of Phthalate Esters (PAEs) and Phthalate Monoesters towards Human Carboxylesterases (CESs). Toxicol. Appl. Pharmacol..

[7] Zhang Y., Yang Y., Tao Y., Guo X., Cui Y., Li Z. (2023). Phthalates (PAEs) and Reproductive Toxicity: Hypothalamic-Pituitary-Gonadal (HPG) Axis Aspects. J. Hazard. Mater..

[8] Zhao Q., Liu Y., Chuo Y., Wang X., Jiao Y., Shi W., Bao Y. (2024). Cuscuta chinensis flavonoids alleviate ovarian damage in offspring female mice induced by BPA exposure during pregnancy by regulating the central carbon metabolism pathway. Ecotoxicol. Environ. Saf..

[9] Zhang Y., Jiao Y., Li Z., Tao Y., Yang Y. (2021). Hazards of Phthalates (PAEs) Exposure: A Review of Aquatic Animal Toxicology Studies. Sci. Total Environ..

[10] Isci G. (2024). Assessment of Phthalate Esters in Packaged Fruit Juices Sold in the Turkish Market and Their Implications on Human Health Risk. Food Chem..

[11] Mikula P., Svobodová Z., Smutná M. (2005). Phthalates: Toxicology and Food Safety—A Review. Czech J. Food Sci..

[12] Arfaeinia L., Dobaradaran S., Nasrzadeh F., Shamsi S., Poureshgh Y., Arfaeinia H. (2020). Phthalate Acid Esters (PAEs) in Highly Acidic Juice Packaged in Polyethylene Terephthalate (PET) Container: Occurrence, Migration and Estrogenic Activity-Associated Risk Assessment. Microchem. J..

[13] Wang R., Ma X., Zhang X., Li X., Li D., Dang Y. (2019). C8-Modified Magnetic Graphene Oxide Based Solid-Phase Extraction Coupled with Dispersive Liquid-Liquid Microextraction for Detection of Trace Phthalate Acid Esters in Water Samples. Ecotoxicol. Environ. Saf..

[14] Jose Varghese R., Zikalala N., Sakho E.H.M., Oluwafemi O.S. (2020). Green Synthesis Protocol on Metal Oxide Nanoparticles Using Plant Extracts, in Colloidal Metal Oxide Nanoparticles.

[15] Keshavarzi M., Ghorbani M., Mohammadi P., Pakseresht M., Ziroohi A., Rastegar A. (2022). Development of a Magnetic Sorbent Based on Synthesis of MOF-on-MOF Composite for Dispersive Solid-Phase Microextraction of Five Phthalate Esters in Bottled Water and Fruit Juice Samples. Microchem. J..

[16] Prokůpková G., Holadová K., Poustka J., Hajšlová J. (2002). Development of a Solid-Phase Microextraction Method for the Determination of Phthalic Acid Esters in Water. Anal. Chim. Acta.

[17] Ghaedi M., Sadeghian B., Pebdani A.A., Sahraei R., Daneshfar A., Duran C. (2012). Kinetics, Thermodynamics and Equilibrium Evaluation of Direct Yellow 12 Removal by Adsorption onto Silver Nanoparticles Loaded Activated Carbon. Chem. Eng. J..

[18] Ahn C.K., Park D., Woo S.H., Park J.M. (2009). Removal of Cationic Heavy Metal from Aqueous Solution by Activated Carbon Impregnated with Anionic Surfactants. J. Hazard. Mater..

[19] Moazzen M., Mousavi Khaneghah A., Shariatifar N., Ahmadloo M., Eş I., Baghani A.N., Yousefinejad S., Alimohammadi M., Azari A., Dobaradaran S. (2019). Multi-Walled Carbon Nanotubes Modified with Iron Oxide and Silver Nanoparticles (MWCNT-FeO/Ag) as a Novel Adsorbent for Determining PAEs in Carbonated Soft Drinks Using Magnetic SPE-GC/MS Method34. Arab. J. Chem..

[20] Zhang S., Wang R., Wu Y., Chen Z., Tong P., He Y., Lin Z., Cai Z. (2022). One-Pot Synthesis of Magnetic Covalent Organic Frameworks for Highly Efficient Enrichment of Phthalate Esters from Fine Particulate Matter. J. Chromatogr. A.

[21] Liu Y., Song W., Zhou D., Han F., Gong X., Pan P. (2022). A New Core-Shell Magnetic Mesoporous Surface Molecularly Imprinted Composite and Its Application as an MSPE Sorbent for Determination of Phthalate Esters. RSC Adv..

[22] Javed R., Zia M., Naz S., Aisida S.O., Ain N., Ao Q. (2020). Role of capping agents in the application of nanoparticles in biomedicine and environmental remediation: Recent trends and future prospects. J. Nanobiotechnology.

[23] Xu R., Gao H.T., Zhu F., Cao W.X., Yan Y.H.M., Zhou X., Xu Q., Ji W.L. (2016). SPE–UPLC–MS/MS for the Determination of Phthalate Monoesters in Rats Urine and Its Application to Study the Effects of Food Emulsifier on the Bioavailability of Priority Controlling PAEs. J. Chromatogr. B.

[24] Meng L., Lan T., Xu J., Zhao P., Lei J. (2024). Adjusting Structure-Activity Relationship to Obtain Hybrid Proton Exchange Membrane with Enhanced Transport Efficiency by Introducing Functionalized Nano-Coated MOFs. J. Membr. Sci..

[25] Cheng L., Huang R., Cao Q., Liu N., Li P., Sun M., Qin H., Wu L. (2023). Magnetic Metal-Organic Frameworks as Adsorbents for the Detection of Azo Pigments in Food Matrices. Food Chem..

[26] Assen A.H., Yassine O., Shekhah O., Eddaoudi M., Salama K.N. (2017). MOFs for the Sensitive Detection of Ammonia: Deployment of Fcu-MOF Thin Films as Effective Chemical Capacitive Sensors. ACS Sens..

[27] Zhao S., Sun Z., Liu H., Zhou Y., Li J., Wang X., Gong B. (2019). Molecularly Imprinted Polymer Coating on Metal-Organic Frameworks for Solid-Phase Extraction of Fluoroquinolones from Water. J. Sep. Sci..

[28] Ke F., Wang L., Zhu J. (2014). Multifunctional Au-Fe_3_O_4_@MOF Core-Shell Nanocomposite Catalysts with Controllable Reactivity and Magnetic Recyclability. Nanoscale.

[29] Fu Q., Xia Z.-Z., Sun X., Jiang H.-L., Wang L.-L., Ai S., Zhao R.-S. (2023). Recent Advance and Applications of Covalent Organic Frameworks Based on Magnetic Solid-Phase Extraction Technology for Food Safety Analysis. TrAC Trends Anal. Chem..

[30] Xu H., Zhu J., Wu X., Cheng Y., Wang D., Cai D. (2023). Recognition and Quantitative Analysis for Six Phthalate Esters (PAEs) through Functionalized ZIF-67@Ag Nanowires as Surface-Enhanced Raman Scattering Substrate. Spectrochim. Acta Part A Mol. Biomol. Spectrosc..

[31] Yan Y., Yang B., Ji G., Lu K., Zhao Z., Zhang H., Xia M., Wang F. (2023). Tunable Zirconium-Based Metal Organic Frameworks Synthesis for Dibutyl Phthalate Efficient Removal: An Investigation of Adsorption Mechanism on Macro and Micro Scale. J. Colloid Interface Sci..

[32] Zhou Q., Guo M., Wu S., Fornara D., Sarkar B., Sun L., Wang H. (2021). Electrochemical Sensor Based on Corncob Biochar Layer Supported Chitosan-MIPs for Determination of Dibutyl Phthalate (DBP). J. Electroanal. Chem..

[33] Gao J., Fan D., Chu Q., Lyu H., Xie Z. (2022). Fabrication of a Novel Surface Molecularly Imprinted Polymer Based on Zeolitic Imidazolate Framework-7 for Selective Extraction of Phthalates. Microchem. J..

[34] Yeganegi A., Fardindoost S., Tasnim N., Hoorfar M. (2024). Molecularly Imprinted Polymers (MIP) Combined with Raman Spectroscopy for Selective Detection of Δ9-Tetrahydrocannabinol (THC). Talanta.

[35] Guo L., Ma X., Xie X., Huang R., Zhang M., Li J., Zeng G., Fan Y. (2019). Preparation of Dual-Dummy-Template Molecularly Imprinted Polymers Coated Magnetic Graphene Oxide for Separation and Enrichment of Phthalate Esters in Water. Chem. Eng. J..

[36] He J., Wang L., Liu H., Sun B. (2024). Recent Advances in Molecularly Imprinted Polymers (MIPs) for Visual Recognition and Inhibition of α-Dicarbonyl Compound-Mediated Maillard Reaction Products. Food Chem..

[37] Wang X., Wang J., Zhao W., Guo R., Cui S., Huang J., Lu J., Liu H., Liu Y. (2024). Integrated Treatment of Tetracycline in Complex Environments with MIPs-Based FeO-CuO-Au Nanocomposites: Selective SERS Detection and Targeted Photocatalytic Degradation342. J. Alloys Compd..

[38] Movlaee K., Ganjali M.R., Norouzi P., Neri G. (2017). Iron-Based Nanomaterials/Graphene Composites for Advanced Electrochemical Sensors. Nanomaterials.

[39] Hou F., Chang Q., Wan N., Li J., Zang X., Zhang S., Wang C., Wang Z. (2022). A Novel Porphyrin-Based Conjugated Microporous Nanomaterial for Solid-Phase Microextraction of Phthalate Esters Residues in Children’s Food. Food Chem..

[40] Gao J.-J., Lang X.-X., Yu Q.-Q., Li H.-Y., Wang H.-J., Wang M.-Q. (2021). Amphiphilic BODIPY-Based Nanoparticles as “Light-up” Fluorescent Probe for PAEs Detection by an Aggregation/Disaggregation Approach. Spectrochim. Acta Part A Mol. Biomol. Spectrosc..

[41] Wu Q., Song Y., Wang Q., Liu W., Hao L., Wang Z., Wang C. (2021). Combination of magnetic solid-phase extraction and HPLC-UV for simultaneous determination of four phthalate esters in plastic bottled juice. Food Chem..

[42] Liu X., Sun Z., Chen G., Zhang W., Cai Y., Kong R., Wang X., Suo Y., You J. (2015). Determination of phthalate esters in environmental water by magnetic Zeolitic Imidazolate Framework-8 solid-phase extraction coupled with high-performance liquid chromatography. J. Chromatogr. A.

[43] Wu Y., Zhou Q., Yuan Y., Wang H., Tong Y., Zhan Y., Sheng X., Sun Y., Zhou X. (2020). Enrichment and sensitive determination of phthalate esters in environmental water samples: A novel approach of MSPE-HPLC based on PAMAM dendrimers-functionalized magnetic-nanoparticles. Talanta.

[44] Luo Y.-B., Yu Q.-W., Yuan B.-F., Feng Y.-Q. (2012). Fast Microextraction of Phthalate Acid Esters from Beverage, Environmental Water and Perfume Samples by Magnetic Multi-Walled Carbon Nanotubes. Talanta.

[45] Jiang L., Niu J., Zhang Y., Liu H., Huang S., Yuan S., Dong G., Bu L., Song D., Zhou Q. (2024). High Enrichment and Sensitive Measurement of Seventeen Phthalates in Beverages with Metal Organic Framework Functionalized Magnetic MXene Nanocomposite Based on Magnetic Solid Phase Extraction Prior to Gas Chromatography-Triple Quadrupole Mass Spectrometry. Sep. Purif. Technol..

[46] Cao X.-L. (2010). Phthalate Esters in Foods: Sources, Occurrence, and Analytical Methods. Compr. Rev. Food Sci. Food Saf..

[47] Martín-Gómez B., Stephen Elmore J., Valverde S., Ares A.M., Bernal J. (2024). Recent Applications of Chromatography for Determining Microplastics and Related Compounds (Bisphenols and Phthalate Esters) in Food. Microchem. J..

[48] Yang R., Liu Y., Yan X., Liu S. (2016). Simultaneous Extraction and Determination of Phthalate Esters in Aqueous Solution by Yolk-Shell Magnetic Mesoporous Carbon-Molecularly Imprinted Composites Based on Solid-Phase Extraction Coupled with Gas Chromatography-Mass Spectrometry. Talanta.

[49] Xu M., Liu M., Sun M., Chen K., Cao X., Hu Y. (2016). Magnetic Solid-Phase Extraction of Phthalate Esters (PAEs) in Apparel Textile by Core-Shell Structured FeO@silica@triblock-Copolymer Magnetic Microspheres34. Talanta.

[50] Lokhat D., Brijlal S., Naidoo D.E., Premraj C., Kadwa E. (2022). Synthesis of Size-and-Shape-Controlled Iron Oxide Nanoparticles via Coprecipitation and In Situ Magnetic Separation. Ind. Eng. Chem. Res..

[51] de Jesús Ruíz-Baltazar Á., Reyes-López S.Y., de Lourdes Mondragón-Sánchez M., Robles-Cortés A.I., Pérez R. (2019). Eco-Friendly Synthesis of FeO Nanoparticles: Evaluation of Their Catalytic Activity in Methylene Blue Degradation by Kinetic Adsorption Models34. Results Phys..

[52] Pathak G., Rajkumari K., Rokhum S.L. (2019). Wealth from Waste: M. Acuminata Peel Waste-Derived Magnetic Nanoparticles as a Solid Catalyst for the Henry Reaction. Nanoscale Adv..

